# The Relation between Resistin (−420C/G) Single Nucleotide Variant, Resistin Serum Concentration, Carbohydrate, and Lipid Parameters and Fried Food Taste Preference in Patients with Hypertriglyceridemia

**DOI:** 10.3390/nu14235092

**Published:** 2022-12-01

**Authors:** Ewa Miller-Kasprzak, Katarzyna Musialik, Matylda Kręgielska-Narożna, Monika Szulińska, Paweł Bogdański

**Affiliations:** Department of Treatment of Obesity, Metabolic Disorders, and Clinical Dietetics, Poznań University of Medical Sciences, Szamarzewskiego 82/84, 60-569 Poznań, Poland

**Keywords:** resistin, single nucleotide variants, hypertriglyceridemia, fried food taste preference, metabolic parameters

## Abstract

Background: Resistin is a proinflammatory adipokine involved in metabolic disorders. Its interplay with hypertriglyceridemia remains to be elucidated. We aimed to evaluate the relationship between resistin (−420C/G) single nucleotide variant (SNV) and metabolic parameters and preference for fried food consumption in hypertriglyceridemia. Methods: The study enrolled 179 hypertriglyceridemic (HTG) and 182 normotriglyceridemic (NTG) patients. Anthropometric measurements, serum resistin, insulin and fasting glucose concentration, a homeostatic model assessment—insulin resistance (HOMA-IR), triglycerides (TG), cholesterol concentration, and fried food taste preference (FP) or other cooking methods preference (OP) were assessed in the study. Genotyping was performed by polymerase chain reaction-restriction fragment length polymorphism. Results: HTG and NTG groups did not differ significantly in serum resistin concentration; HTG individuals demonstrated significantly increased serum levels of TG, glucose, total cholesterol (TCH), and HOMA-IR and decreased HDL cholesterol. Resistin, insulin, glucose, HOMA-IR, and cholesterol fractions were similar among particular resistin genotypes in HTG, NTG, FP, or OP groups. TG and TCH concentrations differ significantly among CG and CC genotypes in the FP group. Considering the FP group, GG and CG genotypes appeared more frequently in hyperlipidemic (OR 2.6 95% CI; 1.16–5.82; *p* = 0.01; significant after Bonferroni correction) than in NTG patients. Multivariable logistic regression models showed that the G allele and CG genotype of SNV (−420C/G), adjusted for selected confounders such as fried food preference, increased the odds of hypertriglyceridemia about twofold. Conclusions: Allele G and CG genotype of resistin SNV (−420C/G) are linked with the preference for fried food taste in hypertriglyceridemic patients.

## 1. Introduction

Hypertriglyceridemia encompasses elevated triglycerides due to several concomitant diseases such as obesity and insulin resistance, pharmacological medication use, or a consequence of a diet rich with high glycemic and saturated fat load [1,2]. Triglycerides appear in the plasma in two main ways: endogenous and related to the synthesis in the liver and very-low-density lipoprotein (VLDL) particles and exogenous as a result of ingested dietary fat. Thus, non-pharmacological therapy for mild hypertriglyceridemia includes lifestyle modification based on diet and physical activity [2,3]. In the Western diet, a lot of dishes are based on fats and vegetable oils and are prepared by the deep-frying method [4]. Frying with added oil leads to the formation of lipid peroxidation products that may modify not only fried food but also a fried medium and even influence amino acid biosynthesis pathways in human gastric cells in vitro or change gene expression [4,5,6]. Fried food consumption may be associated with cardiovascular disease risk (CVD) [7], although some authors did not observe a relationship between the consumption of fried foods and coronary heart disease (CHD) [5]. Nevertheless, the results might depend on the method of frying, oil type, and the sort of food subjected to the frying process [5,8]. Increasing pieces of evidence suggest that the Western diet is associated with disturbed metabolic homeostasis and is able to influence the immunological system and microbiota, leading to inflammation and metabolic diseases. Metaflammation has become a new term that defines a state of inflammation closely related to non-communicable disorders and harmful lifestyle behavior [3]. Metainflammation is driven by the action of multiple factors, including adipokines, cytokines, and hormones [9]. Among recently described adipokines, resistin has been proposed as a proinflammatory link between insulin resistance and obesity [10]. Resistin is a cysteine-rich peptide encoded by the *RETN* gene and expressed mainly by macrophages. Resistin is secreted to the circulation and is believed to act as a proinflammatory factor in several conditions [11,12,13]. Among several single nucleotide sequence variants (SNV) described for *RETN*, some are directly related to the level of *RETN* expression, e.g., SNV (−420C/G) [14]. It has been proposed that the GG genotype of SNV (−420C/G) is accompanied by an increased resistin level as compared to remained genotypes, which was also documented on the epigenetic level [15,16]. The G allele of resistin SNV (−420C/G) has been reported to be linked to non-communicable disorders; however, the results seem inconsistent among studies [13,17,18,19]. Resistin has also been demonstrated to influence serum triglyceride levels [17,18]. Data obtained from Swedish cohorts demonstrated that circular resistin concentration is positively related to unhealthy, e.g., fat-rich, diet and inversely to healthy diet patterns [20]. It has been announced that among six tastes, a new taste modality exists that provides the sense of fat, probably mediated by the CD36 scavenger receptor, a long fatty acids transporter [21,22]. Interestingly, resistin has been reported to upregulate CD36 expression in human macrophages [23]. The role of SNV (−420C/G) in the aspect of hypertriglyceridemia and fried food preference remains unclear.

The goal of our study was to evaluate the association between serum resistin concentration, resistin SNV (−420C/G) frequency, and its putative relation with carbohydrate and lipid parameters and the fried food consuming preference in individuals with elevated triglycerides level.

## 2. Materials and Methods

### 2.1. Study Population

The study was approved by the Local Bioethics commission (Approvals No. 1312/18, No. 359/15) and conducted in agreement with the Helsinki Declaration. Subjects included in the study were in the Metabolic Disorders Outpatient Clinic at the Clinical Hospital of Lord’s Transfiguration in Poznan, Poland. After describing the study goal, each patient that fully accepted the purpose of the study completed a consent form to participate. A total of 179 individuals with hypertriglyceridemia (HTG) and 192 patients with normal triglyceride levels (NTG) were enrolled in the study. For 10 individuals from the NTG group, the genotyping data only were available. The flow diagram of the study is presented in Figure S1. The inclusion criteria were as follows: ≥18 years old, stable body mass (±3 kg) over the previous month (self-reported by patients), and elevated triglycerides (TG) or normal level of TG for the HTG and NTG groups, respectively. Hypertriglyceridemia was defined as a level of TG exceeding 150 mg/dL according to the guidelines of the European Society of Cardiology/European Atherosclerosis Society for the management of dyslipidemias [24]. The exclusion criteria were as follows: type-2 diabetes mellitus, hypertension, any hypolipemic treatment, clinically significant impaired liver function, severe and chronic kidney diseases, severe state of inflammation, pregnancy, tumor disease, and alcohol or nicotine abuse, or any other conditions that, in the opinion of researchers, may influence the results.

### 2.2. Anthropometric and Biochemical Parameters

Each study participant was instructed to maintain a usual diet, not consume alcohol, coffee, or other caffeine-containing products, and not perform intensive physical exercises in the period 24 h prior to the examination. Each patient was asked to respond to their preference for fried food taste or other cooking method food taste in their routine dietary habits. Patients expressed the response on a dichotomous scale (0—other cooking method taste preference) or (1—fried food taste preference). Each patient also responded to the consumption of fried food or food prepared with other methods in their routine diet, expressed on the dichotomous scale (0—other cooking method prepared food) and (1—fried food). OP group included subjects with a 00 code and 11 code having individuals in FP groups, respectively. Individuals with other codes or unsure about their taste preferences and fried food consumption were not included in the above groups.

Patients were examined after an overnight fast, wearing light clothes. The weight and height of each patient in the study were measured with RADWAG WPT 100/200 OW stadiometer with an electric scale of the accuracy of 0.1 kg and 0.5 cm, respectively. The standard equation to body weight (kg)/height (m²) served to calculate BMI. The neck circumference (NC) was measured by the tape from the level underneath the laryngeal prominence perpendicular to the long axis of the neck.

Peripheral blood samples were collected from each patient in the morning during fasting. The serum was separated to estimate particular biochemical parameters. Resistin concentration was evaluated from serum collected samples using an immunoenzymatic method with a commercially available kit (FineTest, Wuhan, China). Remained biochemical parameters such as total cholesterol, TG, high-density lipoprotein (HDL) insulin, and glucose concentration were obtained using routine enzymatic methods in a commercial laboratory. Patients with impaired fasting glucose had oral glucose tolerance test performed and diabetes state was excluded. Low-density lipoprotein (LDL) serum concentration was derived from the Friedewald formula [25]. The value for the homeostasis model assessment of insulin resistance (HOMA-IR) was evaluated by Matthew’s method: fasting insulin (µIU/mL) × fasting glucose (mg/dL)/22.5 [26]. 

### 2.3. Genotyping Analysis

Analysis of *RETN* genotypes was performed as described before [19]. Briefly, genomic DNA was isolated from ethylenediaminetetraacetic acid-containing peripheral blood by Master Pure DNA Purification Kit (Epicentre, Lucigen, Middleton, WI, USA) and amplified in a polymerase chain reaction with forward 5′TGTCATTCTCACCCAGAGACA3′ and reverse 5′TGGGCTCAGCTAACCAAATC3′ primers [27] complementary to the sequences adjacent the site of SNV (−420C/G) (rs1862513) polymorphism (Acc. no. NG_023447) and checked by sequencing. After restriction reaction with *BBsI* restriction endonuclease (Thermo Fisher Scientific, Waltham, MA, USA), digested fragments were visualized on 3% agarose gel with Midori Green (Nippon Genetics, Tokyo, Japan). About 10% of randomly selected samples were re-genotyped to confirm the reproducibility of the assay.

### 2.4. Statistical Analysis

The statistical evaluation was performed by Statistica 13.0 software (Statsoft, Tulsa, OK, USA). Values were presented as a mean with standard deviation (SD). The Shapiro–Wilk test was applied to check the normal distribution of variables. The frequencies of genotypes were estimated by a contingency table analysis chi-squared (*χ*2) test or Fisher test if applicable. The odds ratio (OR) and 95% confidence intervals (95% CI) were assessed. Snphwe package in Python 3.10. was used to check with the Hardy–Weinberg equilibrium. For variables that were in compliance with the Gaussian curve, the Student’s *t*-test and analysis of variance (ANOVA) for unrelated variables were applied. For non-compliance of variables with the Gaussian curve, a Mann–Whitney test and Kruskal–Wallis test for unrelated variables with the post hoc test were applied. For normally distributed values, the Pearson correlation test was used, and Spearman’s rank correlation test was applied for variables not fulfilling the normal distribution to evaluate the correlation between examined parameters. The Bonferroni correction was applied to account for multiple comparisons for analyzed groups. Multivariable logistic regression models were used to determine the influence of resistin SNV (−420C/G) and confounding variables on hypertriglyceridemia phenotype. The power of the study was calculated using Genetic Association Study (GAS) Power Calculator. The level of *p* < 0.05 was considered statistically significant. 

## 3. Results

Individuals from HTG and NTG groups differed significantly in TG levels (262.53 ± 147.09 vs. 104.42 ± 28.03; *p* < 0.0000001). Several metabolic parameters increased in the HTG group as compared to NTG individuals, among them fasting glucose (*p* < 0.003), total cholesterol (*p* < 0.0002), and HOMA-IR value (*p* < 0.04). The concentration of HDL cholesterol was significantly elevated in the NTG group (*p* < 0.000001) compared to in HTG participants. Both groups revealed similar age distribution. In the NTG group, significant female sex prevalence (*p* < 0.01) was observed. Both the presence of obesity and frying method preference percentages were similar between the studied groups. The characteristics of the population are summarized in Table 1.

The genotype distribution for SNV (−420C/G) in the studied population was consistent with Hardy–Weinberg equilibrium (*p* = 0.36), observed allele frequencies (0.34 for G and 0.66 for C allele) were comparable to the mean allele frequency (MAF) TopMed population stated in the Ensembl database for the rs1862513 variant. The power of the case-control study was calculated for 0.84 in the dominant model of inheritance with alpha = 0.05. 

To carry out further analysis, we split individuals into HTG and NTG groups according to the presence or absence of elevated TG concentration and FP and OP groups according to the fried food taste preference. To analyze the dominant model of inheritance, we grouped studied participants into the GG + CG and CC genotype-carrying individuals.

The observed frequency of SNV (−420C/G) genotypes did not differ significantly between HTG and NTG individuals nor according to the additive or dominant model of inheritance. We obtain comparable, not significant results regarding the distribution of reistin genotypes and genotype groups between FP and OP participants. Considering the FP group alone, HTG individuals from the FP group possessed GG and CG genotypes more frequently (OR 2.6 95% CI; 1.16–5.82; *p* = 0.01, significant after Bonferroni correction). OP group did not differ significantly in the frequency of particular resistin genotypes or genotype groups among HTG and NTG patients. Similar results were obtained in the HTG group when compared according to cooking preference. With respect to the NG group considered alone, we observed that GG + CG genotypes were less frequent in the FP group (OR 0.47; 0.23–0.98; *p* = 0.04) but became statistically insignificant after Bonferroni correction. The results of the distribution of SNV (−420C/G) in studied individuals are summarized in Table 2.

The serum resistin concentration compared among genotypes and GG+ CG vs. CC subjects in each HTG, NTG, OP, and FP group did not differ significantly (Table 3.). We noticed the same results considering other studied carbohydrate and lipid parameters in HTG, NTG, and OP groups (Table 4 and Table 5). Conversely, the FP group belonging subjects presented significantly higher triglyceride concentration while carrying the CG genotype compared to CC genotype-bearing individuals (*p* = 0.02; Table 3). The result became insignificant after Bonferroni correction. Moreover, in the GG + CG group, the concentration of triglycerides was significantly elevated (and remained significant after Bonferroni correction) compared to CC genotype carriers (*p* = 0.0006; Table 3). Patients from the GG + CG group that preferred the frying taste of food also demonstrated significantly higher total cholesterol concentration (*p* = 0.04) than their CC genotype counterparts (Table 5), but results became insignificant after Bonferroni correction.

Studied parameters revealed several correlations that were observed in both GG + CG and CC genotype-carrying individuals.

In the whole population, we noticed the correlations between resistin concentration and insulin (r = 0.45, *p* = 0.000000), HOMA-IR value (r = 0.40, *p* = 0.000000), triglycerides correlated with glucose (r = 0.22, *p* = 0.000037), HDL (r = −0.33, *p* = 0.000000), HOMA-IR (r = 0.15, *p* = 0.010409; not significant after Bonferroni correction) and TCH concentration (r = 0.24; *p* = 0.000002). 

In the group carrying CG and CG genotypes, resistin concentration correlated with HOMA-IR (r = 0.32, *p* = 0.000144), insulin (r = 0.36, *p* = 0.000011), and triglycerides correlated with glucose (r = 0.18, *p* = 0.015882; not significant after Bonferroni correction ), HDL (r = −0.32, *p* = 0.000003), and TCH (r = 0.23, *p* = 0.000985; not significant after Bonferroni correction). 

Concerning GG + CG possessing individuals from the HTG group, we noticed correlations between resistin, HOMA-IR (r = 0.37, *p* = 0.000879), and insulin (r = 0.41, *p* = 0.000174) and between triglycerides and HDL (r = −0.36, *p* = 0.000161). In respective NTG individuals, the correlations between resistin and HDL (r = 0.36, *p* = 0.005585; not significant after Bonferroni correction), HOMA-IR (r = 0.27, *p* = 0.039224; not significant after Bonferroni correction), and insulin (r = 0.34, *p* = 0.008120; not significant after Bonferroni correction) were documented. 

In the FP group possessing GG + CG genotypes, resistin correlated with triglycerides (r = 0.33, *p* = 0.034394; not significant after Bonferroni correction), HOMA-IR (r = 0.48; *p* = 0.000436), and insulin (r = 0.47; *p* = 0.000543) and triglycerides correlated with HDL (r = −0.41, *p* = 0.002373; not significant after Bonferroni correction) and TCH (r = 0.42, *p* = 0.001712; not significant after Bonferroni correction). Concerning the respective OP group, we noticed the correlation of resistin with HOMA-IR (r = 0.35, *p* = 0.000924; not significant after Bonferroni correction) and insulin concentration (r = 0.40, *p* = 0.000967; not significant after Bonferroni correction) and triglycerides with HDL (r = −0.23; *p* = 0.005725; not significant after Bonferroni correction) and TCH (r = 0.18; *p* = 0.030878; not significant after Bonferroni correction).

In the whole group carrying the CC genotype, we documented correlations of resistin and HOMA-IR (r = 0.49, *p* = 0.000000) and insulin concentration (r = 0.53, *p* = 0.000000) and triglycerides with glucose (r = 0.26, *p* = 0.001280; not significant after Bonferroni correction), HDL (r = −0.38, *p* = 0.000000) and TCH concentration (r = 0.19, *p* = 0.002289; not significant after Bonferroni correction ). 

In HTG individuals harboring CC genotype, the correlations between resistin and HOMA-IR (r = 0.62, *p* = 0.000000) and insulin (r = 0.79, *p* = 0.000000) were present, and triglycerides correlated with glucose (r = 0.32, *p* = 0.004972; not significant after Bonferroni correction) and HDL concentration (r = −0.32, *p* = 0.003608; not significant after Bonferroni correction). 

In NTG participants possessing the CC genotype, the correlations between resistin and HOMA-IR (r = 0.39, *p* = 0.001541; not significant after Bonferroni correction) and insulin (r = 0.33, *p* = 0.008799; not significant after Bonferroni correction) were present. 

FP group carrying CC genotype revealed the correlations between resistin and HOMA-IR (r = 0.52, *p* = 0.000184) and insulin (r = 0.53, *p* = 0.000113) along with the correlation of triglycerides with HDL (r = −0.39, *p* = 0.002754; not significant after Bonferroni correction). In respective OP individuals, resistin correlated with HOMA-IR (r = 0.49, *p* = 0.000013) and insulin (r = 0.52, *p* = 0.000003) and triglycerides correlated with glucose (r = 0.30, *p* = 0.003431; not significant after Bonferroni correction), HDL (r = −0.38, *p* = 0.000038), and TCH (r = 0.23, *p* = 0.011609; not significant after Bonferroni correction). All statistically significant correlations noticed in the study were summarized in Table 6.

We applied three multivariable logistic regression models to evaluate the risk of hypertriglyceridemia in relation to the studied resistin variant (Table S1). The first model was adjusted for age, gender, and body mass index and showed that the occurrence of the G allele of SNV (−420C/G) significantly increased the risk of hypertriglyceridemia (OR 1.74 CI 95%; 1.08–2.81; *p* = 0.02) and that male gender serves as an independent predictor of hypertriglyceridemia status in this model (OR 2.08 CI 95%; 1.24–3.49; *p* = 0.005). In the second model adjusted for gender, resistin genotypes, fried food preference, and total cholesterol concentration, individuals with CG (vs. CC genotype) have 71% more odds of having hypertriglyceridemia (OR 1.71 CI 95%; 1.05–2.79; *p* = 0.02). Fried food preference independently increases the odds of hypertriglyceridemia in this model (OR 1.95 CI 95%; 1.18–3.21; *p* = 0.008). The second model of regression was additionally adjusted for HOMA-IR value and became the third model where: male gender (OR 2.48 CI 95%; 1.35–4.55; *p* = 0.003), HOMA-IR (OR 1.24 CI 95%; 1.01–1.53; *p* = 0.04) and CG genotype (OR 1.93 CI 95%; 1.05–3.58; *p* = 0.03) remained predictors of hypertriglyceridemia, while fried food preference prediction becomes statistically insignificant (OR 1.46 CI 95%; 0.81–2.64; *p* = 0.2) in this model.

## 4. Discussion

Metabolic disorders are closely linked with several pathological processes, including lipid and carbohydrate pattern abnormalities, low-grade inflammation, and endothelial dysfunction, that disturb physiological homeostasis. Atherogenic dyslipidemia is associated with insulin resistance and is characterized by elevated serum triglyceride concentration and HDL diminished level; thus, it is strongly linked with the risk of CVD [1,28]. A Western diet that is rich in fat and oil-fried dishes is closely connected with metabolic disorders [3]. Insulin regulates VLDL hepatic secretion by diminishing fatty acid influx into the liver and promoting the posttranslational degradation of apolipoprotein B [28]. The name resistin derives from its ability to influence insulin action. 

Our study presents, for the first time to the best of our knowledge, that resistin SNV (−420C/G) G allele is associated with hypertriglyceridemia and moreover, in subjects that prefer consuming fried dishes, those with elevated triglycerides possessed GG and CG genotypes more frequently compared to NTG group. In the FP group, triglyceride concentration was significantly elevated in the G allele having individuals compared to the CC genotype after Bonferroni correction. Located in the promoter region, SNV (−420C/G) resistin variant belongs to functional polymorphism; thus, it affects the expression level of the *RETN* gene. The regulation of resistin expression depends on the genotype and undergoes via binding of specific transcription factors to the promoter region and epigenetically via altering methylation status [14,16]. Menzaghi et al., in the Italian cohort study, confirmed that serum resistin concentration in 70% is heritable [29]. Osawa et al. reported that individuals with the GG genotype are characterized by the highest resistin concentration compared to CG and followed by the CC genotype [15]. Resistin plasma or serum concentration has been studied as a causative factor in several diseases often associated with elevated triglycerides, but the results remain inconsistent [15,30]. In our study, both in the HTG group characterized by elevated triglycerides levels and in NTG individuals, the resistin concentration was comparable. Serum resistin concentration was also comparable among particular GG + CG vs. CC groups and genotypes in our study. Similar results were documented by others [29]. Takshid et al. presented a correlation between serum resistin and triglycerides in studied subjects that stayed in agreement with the uncorrected result in our FP group [17]. However, after Bonferroni correction, our correlation became statistically insignificant; thus it is difficult to compare it with the uncorrected results of the above authors [17]. El Shal et al. performed a study on Egyptian obese individuals with and without glucose intolerances and proved that triglycerides level significantly differed between resistin genotypes and was elevated in obese G allele carriers compared to the CC genotype-having individuals, closely similar to our observation from the FP group that remained statistically significant after Bonferroni correction. The authors also reported an increased total cholesterol level in the GG group compared to CC individuals, similar to our observation from the FP group. However, after Bonferroni correction our result became statistically insignificant same as Bonferroni corrected correlation between serum resistin concentration and triglycerides. El Shal documented significant correlations between serum resistin concentration and triglycerides and HOMA-IR value; however, authors did not apply multiple testing correction to their results [18]. In our study, HOMA-IR value correlated with resistin concentration in the studied population and subgroups of individuals. Menzaghi et al., in the study performed on nondiabetic subjects with normal triglyceride levels, also documented the correlation between serum resistin levels and HOMA-IR in the entire studied population, which was in line with the results from our study [29]. We also showed in the multivariable logistic regression model that adjusted for several confounders that HOMA-IR value was, along with CG genotype, an independent predictor of hypertriglyceridemia in the studied population. Nakashima et al. in their study analyzed the resistin variant in Japanese type 2 diabetic patients with and without a history of CVD as a case-control study. Among other clinical parameters, cases have significantly elevated triglycerides and resistin concentrations compared to controls [13]. No significant correlations between resistin and HOMA-IR, glucose, insulin, or triglyceride levels were observed in this study. Authors found that patients who carried G allele are characterized by high serum resistin levels [13]. Osawa et al. demonstrated that T2DM subjects harboring GG genotype of SNV (−420C/G) had increased serum resistin levels due to the alteration of resistin gene promoter activity [14] and plasma resistin rise gradually CC followed by CG to GG genotype [15]. In another work, Osawa et al. studied Japanese T2M diabetes individuals and found that the serum resistin was elevated in patients with high TG concentrations [30]. Moreover, Osawa et al. confirmed that serum resistin correlated significantly with triglycerides and several factors related to metabolic syndrome in the studied cohort [30]. G allele-carrying morbidly obese patients were also characterized by elevated serum resistin levels in the De Luis study [31]. The serum resistin levels presented no significant association with the resistin variant in the obese and non-obese Tunisian population, and no correlations between resistin level and triglycerides and HOMA-IR value were observed in this study [32]. A pilot study by Makino et al. demonstrated that GG genotype adjusted for confounding factors was an independent predictor of the fasting glucose and HOMA-IR value decrease in T2M patients treated with pioglitazone but serum resistin concentration remained highest in patients harboring the GG genotype, followed by individuals with CG and CC genotypes [33]. It has been found that TGs concentration is gender-dependent [28]. In the Framingham Offspring Study, mean concentrations of plasma TG in men were significantly higher than in women in a population of comparable age to our studied individuals [34]. We confirmed in the logistic regression models that men’s gender adjusted for other confounders independently from the G allele of SNV (−420C/G) increased the risk of developing hypertriglyceridemia. Taking together the data from above studies, the discrepancies among particular results are expected, thus may derive from some reasons such as detailed clinical and biochemical characteristics of studied populations, the exact type of disease chosen as a design for the case-control study, ethnicity of population and finally environmental factors considered to influence the results. 

In our study, significant results considered the group of individuals that have elevated triglycerides and prefer frying dishes. Moreover, in our logistic regression model, a preference to choose fried dishes adjusted for confounders was, along with CG genotype, an independent factor for developing elevated triglyceride levels. However, in this model of regression, after adjusting for HOMA-IR value, the preference for fried food became insignificant, although CG genotype remained an independent predictor of hypertriglyceridemia. The study by Luis et al. on obese Caucasian patients subjected to a low-fat hypocaloric diet demonstrated that glucose, total insulin cholesterol, and HOMA-IR after a 3-month diet decreased greater in GG allele-possessing individuals compared to the CC + CG group [35]. Thus, the glucose and lipid pathways are biochemically linked, which implicated linked changes in clinical lipid and carbohydrate parameters, particularly in patients with metabolic disorders [36]. It has been documented that a high-fat diet influences the microbiome and switches toward phenotype characteristics of obesity and metabolic disorders [37]. The experiment performed on rats fed with pork fat cooked with different methods found that deep-fried pork fat increased glucose concentration, triglycerides, and total cholesterol level compared to control rats fed with oils and influence the abundance of Bacteroidetes species [37]. The oil frying process leads to lipid peroxidation. Compounds derived from this process, such as water-soluble aldehydes, may modify gene expression [6]. The Western diet includes several nutrient components with proinflammatory potential [3]. Systemic inflammation has been suggested to be engaged in the concentration of resistin in circulation [12]. Lemming et al. performed a study on large Swedish cohorts that analyzed healthy and unhealthy dietary patterns associations with 21 protein biomarkers and found resistin to be inversely associated with a healthy diet pattern but related positively with Western-type dietary patterns [20]. In a large cross-sectional study of random adults, Leon et al. showed a positive correlation between serum resistin level and saturated dietary fat intake and a negative correlation between monosaturated fat intake and adherence to the Mediterranean diet. Authors in multivariate regression models confirmed positive correlations between serum resistin concentration and saturated fat intake, and serum triglyceride concentration in the general population [38]. 

Some studies suggest that resistin may be engaged in appetite regulation [39,40,41]. Tovar et al., in the study on rats, demonstrated that centrally administrated resistin exerts an anorectic effect on food intake in studied animals and observed expression of resistin mRNA in the accurate nucleus of the hippocamp [40]. Resistin mRNA was detected in vitro in a mouse line derived from an *N*-1 hypothalamic neuronal cells suggesting local autocrine and paracrine action of resistin in the hypothalamic region of the brain [41]. Brown et al. demonstrated that resistin influence the expression of a fasting-induced adipose factor in the *N*-1 cell line [41]. 

There are known modalities of taste, among them sour, bitter, sweet, salty, and umami. The announced six-taste quality regards the perception of fat from the diet [21]. The most recent study by Meng et al. performed on a large cohort of Quebec adults showed that CD36 genetic variants are related to dietary fat intake, fat taste preference, and elevated triglyceride concentration [22]. He et al. reported that resistin could regulate B oxidation of fatty acids via action on CD36, a fatty acids transporter [42]. Xu et al. found that resistin modulates the transcript and protein expression of CD36 and promotes the accumulation of lipids in human macrophages [23]. Similarly, another study revealed that skeletal muscle cells incubated with resistin also decreased fatty acid uptake along with diminishing CD36 expression [43]. The study performed by Viana et al. in human monocytes (THP-1 line) demonstrated that sunflower-derived soluble aldehydes increase the expression of CD36 on the monocyte cell surface [6]. The measurable level of resistin in human saliva was reported by Mamali et al. in a study conducted on healthy adult volunteers [44]. Geloen et al. demonstrated that CD36 inhibitors can reduce lipid intake and plausibly affect postprandial hypertriglyceridemia [45]. Although resistin was reported to influence CD36 expression, to the best of our knowledge, there are so far no studies in the literature regarding the relation between CD36 genetic variants and resistin (−420C/G) SNV.

The limitation of our study includes the relatively low number of study participants; however, our aim was more to evaluate the impact of resistin variant on the concentrations of lipid and carbohydrate parameters and not to solely evaluate the distribution of genetic variants in a large cohort. Moreover, the observational character of the study does not allow us to assess the cause-and-effect association. Concerning preference for frying food among studied individuals, more detailed data could be harvested, such as the usual type of fat used for frying or re-usage of the fat. However, such approach requires a larger population to properly study detailed associations. 

According to nutrigenetics, nucleotide variants occurrence can predispose individuals to particular food preferences and reactions. Recent studies demonstrated that sequence variants of genes not directly related to classical taste receptors could be associated with the consumption of unhealthy food and abnormal triglyceride concentration in studied patients [22,46]. Our previous study showed that resistin SNV (−420C/G) is related to salt taste preferences [19]. Here we show that resistin SNV (−420C/G) is linked with hypertriglyceridemia, selected metabolic parameters, and the preference to consume fried food. However, the exact mechanism of resistin action remains to be elucidated in the future, preferably through interventional studies to provide causal conclusions. 

## Figures and Tables

**Table 1:** Clinical characteristics of the study population.

Parameter	*N*	Hypertriglyceridemia (HTG) *n* = 179	Normotriglyceridemia (NTG) *n* = 182	*p*-Value
Female %		59.89	71.66	<0.01 ^^^
Age (years)	179/182	60.21± 11.19	58.34± 12.16	NS *
Body mass (kg)	179/182	87.26 ± 13.20	84.96 ± 18.53	NS ^#^
BMI (kg/m²)	179/182	31.59 ± 3.82	29.81 ± 5.79	NS *
Neck circumference (cm)	179/182	39.35 ± 6.42	37.99 ± 9.30	NS *
Glucose (mg/dL)	172/143	107.94 ± 37.52	97.37 ± 23.71	<0.003 *
Insulin (mg/dL)	135/119	14.05 ± 13.64	13.30 ± 6.82	NS *
HOMA-IR	135/119	3.75 ± 3.49	3.06 ± 1.49	0.04 *
Total cholesterol (mg/dL)	165/172	209.60 ± 42.46	194.39 ± 36.56	<0.0002 *
LDL cholesterol (mg/dL)	165/172	116.40 ± 59.89	110.78 ± 31.30	NS *
HDL cholesterol (mg/dL)	179/182	54.90 ± 12.83	62.34 ± 14.89	<0.000001 *
Resistin (ng/mL)	134/119	7.65 ± 3.87	7.61 ± 3.73	NS *
TG (mg/dL)	179/182	262.53 ± 147.09	104.42 ± 28.03	<0.0000001 ^#^
Obesity %		52.24	44.76	NS ^^^
Frying preference %		38.73	29.93	NS ^^^

SD: standard deviation; *p*: statistical significance; NS: not significant; *N*: (number of patients), BMI: body mass index; HOMA-IR: homeostatic model assessment-insulin resistance; LDL: low-density lipoprotein; HDL: low-density lipoprotein; TG: triglycerides; TCH: total cholesterol. * Mann–Whitney U test; ^#^ *t*-Student test. ^ χ^2^ test. All other values are expressed as mean ± SD or percentage.

**Table 2:** Genotype frequencies of SNV (−420C/G) among: hypertriglyceridemic (HTG) and normoglicerydemic (NTG) individuals, frying preference (FP), and other cooking methods preference (OP) subjects.

Genotype	Hyper Triglyceridemia (HTG)			Normo Triglyceridemia (NTG)	Frying Preference (FP)			Other Cooking Preference (OP)
CC	74 (41.44%)			93 (48.44%)	52 (48.15%)			107 (43.85%)
CG	83(46.37%)			75 (39.06%)	36 (44.22%)			110 (45.08%)
GG	22 (12.29%)			24 (12.50%)	18 (13.63%)			27 (10.89%)
Comparison	χ^2^	*p*-value	OR	(95% CI)	χ^2^	*p*-value	OR	(95% CI)
CC/CG/GG	2.20	0.33	-	-	4.64	0.09	-	-
GG + CG/CC	1.88	0.16	1.33	(0.88–2.00)	0.8	0.36	0.81	(0.51–1.23)
	Hyper triglyceridemia (HTG)					Frying Preference (FP)		
Genotype	Frying preference (FP)			Other cooking preference (OP)	Hyper triglyceridemia (HTG)			Normo triglyceridemia (NTG)
CC	26 (40.00%)			46 (42.20%)	26 (40.00%)			26 (63.41%)
CG	26 (40.00%)			54 (49.54%)	26 (40.00%)			10 (24.39%)
GG	13 (20.00%)			9 (8.26%)	13 (20.00%)			5 (12.20 %)
Comparison	χ^2^	*p*-value	OR	(95% CI)	χ^2^	*p*-value	OR	(95% CI)
CC/CG/GG	5.29	0.07	-	-	5.51	0.06		
GG + CG/CC	0.08	0.77	1.09	(0.58–2.04)	5.51	0.01 ^a^	2.6	(1.16–5.82)
	Normo triglyceridemia (NTG)					Other cooking preference (OP)		
Genotype	Frying preference (FP)			Other cooking preference (OP)	Hyper triglyceridemia (HTG)			Normo trigliceridemia (NTG)
CC	26 (63.41%)			61(45.19%)	46 (42.20%)			61 (45.19%)
CG	10 (24.39%)			56(41.48%)	54 (49.54%)			56 (41.48%)
GG	5 (12.20%)			18 (13.33%)	9 (8.26%)			18 (13.33%)
Comparison	χ^2^	*p*-value	OR	(95% CI)	χ^2^	*p*-value	OR	(95% CI)
CC/CG/GG	4.59	0.10	-	-	2.39	0.30	-	-
GG + CG/CC	4.18	0.04 ^b^	0.47	(0.23–0.98)	0.21	0.64	1.13	(0.68–1.88)

*p*—statistical significance; OR—odds ratio; CI—confidence interval; χ^2^ test. ^a^ significant after Bonferroni correction (*p* < 0.0125); ^b^ Not significant after Bonferroni correction (*p* > 0.0125).

**Table 3:** Serum concentration of resistin and triglycerides compared among SNV (−420C/G) genotypes and genotype groups in the studied population.

Parameter	Genotype	Total	Hyper Triglyceridemia (HTG)	Normo Triglyceridemia (NTG)	Frying Preference (FP)	Other Cooking Preferences (OP)
Resistin (ng/mL)	CC	7.24 ± 2.89 112	7.11 ± 2.29 54	7.33 ± 3.38 58	7.03 ± 3.08 44	7.41 ± 2.80 67
	CG	8.12 ± 4.6 110	8.48 ± 5.07 64	7.83 ± 3.92 46	7.55 ± 4.32 31	7.99 ± 4.26 76
	GG	7.13 ± 3.31 31	6.45 ± 1.35 16	7.99 ± 4.56 15	6.90 ± 1.53 14	7.49 ± 4.49 16
Comparison	CC/CG/GG	*p* = 0.54 **	*p* = 0.39 **	*p* = 0.71 **	*p* = 0.87 **	*p* = 0.76 **
	GG + CG/CC	*p* = 0.50 *	*p* = 0.34 *	*p* = 0.64 *	*p* = 0.70 *	*p* = 0.98 *
TG (mg/dL)	CC	172.63 ± 103.77 161	252.39 ± 151 74	105.72 ± 26.65 87	160.65 ± 69.22@ 52	176.38 ± 116.402 108
	CG	193.25 ± 165.09 154	276.54 ± 191.47 83	101.07 ± 29.32 71	258.60 ± 229.72@ 38	173.60 ± 135.401 112
	GG	170.79 ± 76.39 46	237.50 ± 151.47 22	109.65 ± 29.04 24	201.38 ± 64.43 18	151.39 ± 79.50 27
Comparison	CC/CG/GG	*p* = 0.66 ^##^	*p* = 0.89 ^##^	*p* = 0.43 ^##^	*p* = 0.01 ^## b^ *p* = 0.02@	*p* = 0.72 ^##^
	GG + CG/CC	*p* = 0.31 ^#^	*p* = 0.55 ^#^	*p* = 0.74 ^#^	*p* = 0.0006 ^# a^	*p* = 0.77 ^#^

TG: triglycerides. * Mann–Whitney U test; ^#^ *t*-Student test; ^##^ analysis of variance (ANOVA), ** Kruskal–Wallis ANOVA; @ post-hoc test; All other values are expressed as mean ± SD. ^a^ Significant after Bonferroni correction (*p* < 0.0031); ^b^ Not significant after Bonferroni correction (*p* > 0.0031).

**Table 4:** Serum concentration of carbohydrate parameters compared among SNV (−420C/G) genotypes and genotype groups in the studied population.

Parameter	Genotype	Total	Hyper Triglyceridemia (HTG)	Normo Triglyceridemia (NTG)	Frying Preference (FP)	Other Cooking Preferences (OP)
Glucose (mg/dL)	CC	101.21 ± 27.69 140	106.70 ± 33.78 70	95.82 ± 18.81 70	102.72 ± 31.09 51	100.56 ± 25.95 88
	CG	106.49 ± 37.05 135	110.90 ± 41.31 80	99.85 ± 30.20 55	104.05 ± 19.14 37	106.76 ± 41.87 95
	GG	99.72 ± 30.22 40	102.95 ± 37.47 22	95.77 ± 18.18 18	103.55 ± 41.67 18	97.19 ± 16.50 21
Comparison	CC/CG/GG	*p* = 0.19 **	*p* = 0.34 **	*p* = 0.92 **	*p* = 0.21 **	*p* = 0.76 **
	GG + CG/CC	*p* = 0.41 *	*p* = 0.66 *	*p* = 0.79 *	*p* = 0.39 *	*p* = 0.61 *
Insulin (mg/dL)	CC	13.00 ± 4.90 112	13.29 ± 15.30 54	12.76 ± 4.56 58	12.45 ± 5.14 44	13.50 ± 4.77 67
	CG	14.47 ± 15.40 111	15.47 ± 19.12 65	13.19 ± 8.42 46	12.46 ± 3.39 31	15.57 ± 18.53 77
	GG	13.58 ± 6.32 31	11.49 ± 2.59 16	15.73 ± 8.44 15	13.03 ± 5.64 14	13.99 ± 7.38 16
Comparison	CC/CG/GG	*p* = 0.96 **	*p* = 0.47 **	*p* = 0.76 **	*p* = 0.77 **	*p* = 0.86 **
	GG + CG/CC	*p* = 0.79 *	*p* = 0.88 *	*p* = 0.68 *	*p* = 0.79 *	*p* = 0.95 *
HOMA -IR	CC	3.22 ± 1.43 111	3.54 ± 1.74 54	2.93 ± 1.02 58	3.07 ± 1.46 43	3.34 ± 1.42 67
	CG	3.70 ± 3.80 109	4.19 ± 4.78 65	3.08 ± 1.84 46	3.21 ± 1.06 30	3.92 ± 4.54 76
	GG	3.24 ± 1.44 31	2.95 ± 1.05 16	3.54 ± 1.76 15	3.33 ± 1.47 14	3.18 ± 1.50 16
Comparison	CC/CG/GG	*p* = 0.93 **	*p* = 0.44 **	*p* = 0.72 **	*p* = 0.49 **	*p* = 0.70 **
	GG + CG/CC	*p* = 0.80 *	*p* = 0.73 *	*p* = 0.74 *	*p* = 0.32 *	*p* = 0.68 *

HOMA-IR: homeostatic model assessment-insulin resistance. * Mann–Whitney U test; ** Kruskal–Wallis ANOVA. All other values are expressed as mean ± SD.

**Table 5:** Serum concentration of remained lipid parameters compared among SNV (−420C/G) genotypes and genotype groups in the studied population.

Parameter	Genotype	Total	Hyper Triglyceridemia (HTG)	Normo Triglyceridemia (NTG)	Frying Preference (FP)	Other Cooking Preferences (OP)
TCH (mg/dL)	CC	204.00 ± 76.55 144	220.05 ± 103.16 64	192.49 ± 41.46 80	208.00 ± 87.95 47	202.05 ± 71.53 96
	CG	203.34 ± 64.67 147	206.51 ± 54.55 79	200.65 ± 76.71 68	211.33 ± 51.95 36	199.34 ± 69.28 108
	GG	213.00 ± 41.55 46	212.14 ± 36.76 22	213.76 ± 137.60 24	208.47 ± 36.98 17	217.49 ± 44.40 27
Comparison	CC/CG/GG	*p* = 0.15 **	*p* = 0.88 **	*p* = 0.11 **	*p* = 0.22 **	*p* = 0.10 **
	GG + CG/CC	*p* = 0.17 *	*p* = 0.84 *	*p* = 0.17 *	*p* = 0.04 *^,b^	*p* = 0.56 *
LDL cholesterol (mg/dL)	CC	112.00 ± 46.92 144	116.20 ± 61.39 64	109.85 ± 30.67 80	108.19 ± 33.26 52	115 ± 52.30 106
	CG	115.36 ± 52.20 147	120.58 ± 65.22 79	110.63 ± 35.23 68	114.39 ± 53.75 33	112.10 ± 48.15 109
	GG	110.70± 25.92 46	106.50 ± 3117 22	114.55 ± 19.89 24	107.16 ± 27.99 18	113.78 ± 24.84 27
Comparison	CC/CG/GG	*p* = 0.83 **	*p* = 0.93 **	*p* = 0.62 **	*p* = 0.95 **	*p* = 0.67 **
	GG + CG/CC	*p* = 0.65*	*p* = 0.77 *	*p* = 0.31 *	*p* = 0.82 *	*p* = 0.79 *
HDL cholesterol mg/dL	CC	58.43 ± 13.43 161	53.54 ± 11.11 74	62.41 ± 13.91 87	59.13 ± 14.80 52	58.09 ± 12.81 108
	CG	59.76 ± 16.52 154	55.41 ± 14.36 83	62.51 ± 15.57 71	59.31 ± 18.04 38	59.96 ± 15.56 112
	GG	58.64 ± 14.68 46	55.40 ± 11.44 22	61.60 ± 16.82 24	57.55 ± 11.57 18	59.49 ± 16.83 27
Comparison	CC/CG/GG	*p* = 0.89 **	*p* = 0.77 **	*p* = 0.85 **	*p* = 0.85 **	*p* = 0.74 **
	GG + CG/CC	*p* = 0.95 *	*p* = 0.56 *	*p* = 0.68 *	*p* = 0.18 *	*p* = 0.38 *

LDL: low-density lipoprotein; HDL: low-density lipoprotein; TCH: total cholesterol. * Mann–Whitney U test; ** Kruskal–Wallis ANOVA. All other values are expressed as mean ± SD. ^b^ Not significant after Bonferroni correction (*p* > 0.0031).

**Table 6:** Significant correlations between resistin, TG, HOMA-IR, and other parameters evaluated in the study.

Parameter	Groups	Total	Hyper Triglyceridemia (HTG)	Normo Triglyceridemia (NTG)	Frying Preference (FP)	Other Cooking Preferences (OP)
Resistin (ng/mL)	Both	HOMA-IR (r = 0.40, *p* = 0.000000) Ins (r = 0.45 *p* = 0.000000)	HOMA-IR (r = 0.49, *p* = 0.000000) Ins (r = 0.58, *p* = 0.000000)	HOMA-IR (r = 0.31, *p* = 0.000470) Ins (r = 0.34 *p* = 0.000131)	HOMA-IR (r = 0.50, *p* = 0.000001) Ins (r = 0.52, *p* = 0.000000)	HOMA-IR (r = 0.41, *p* = 0.000000) Ins (r = 0.46, *p* = 0.000000)
	GG + CG	HOMA-IR (r = 0.32, *p* = 0.000144) Ins (r = 0.36, *p* = 0.000011)	HOMA-IR (r = 0.37, *p* = 0.000879) INS (r = 0.41, *p* = 0.000174)	HDL (r = 0.36, *p* = 0.005585 ^b^) HOMA-IR (r = 0.27, *p* = 0.039224 ^b^) Ins (r = 0.34, *p*= 0.008120 ^b^)	TG (r = 0.33, *p* = 0.034394 ^b^) HOMA-IR (r = 0.48, *p* = 0.000436) Ins (r = 0.47, *p* = 0.000543)	HOMA-IR (r = 0.35, *p* = 0.000924 ^b^) Ins (r = 0.40, *p* = 0.000967 ^b^)
	CC	HOMA-IR (r = 0.49, *p* = 0.000000) Ins (r = 0.53, *p* = 0.000000)	HOMA-IR (r = 0.62, *p* = 0.000000) Ins (r = 0.79, *p* = 0.000000)	HOMA-IR (r = 0.39, *p* = 0.001541 ^b^) Ins (r = 0.33 *p* = 0.008799)	HOMA-IR (r = 0.52, *p* = 0.000184) Ins (r = 0.53, *p* = 0.000113)	HOMA-IR (r = 0.49, *p* = 0.000013) Ins (r = 0.52, *p* = 0.000003)
TG (mg/dL)	Both	Glucose (r = 0.22, *p* = 0.000037) HDL (r = −0.33, *p* = 0.000000) TCH (r = 0.24, *p* = 0.000002) HOMA-IR (r = 0.15, *p* = 0.010409 ^b^)	Glucose (r = 0.21 *p* = 0.004589 ^b^) HDL (r = −0.31 *p* = 0.000012) TCH (r = 0.15 *p* = 0.041846 ^b^)	-	HDL (r = −0.37, *p* = 0.000045) TCH (r = 0.34, *p* = 0.000199)	Glucose (r = 0.28, *p* = 0.000042) HDL (r = −0.29, *p* = 0.000001) TCH (r = 0.21, *p* = 0.000001)
	GG + CG	Glucose (r = 0.18, *p* = 0.015882 ^b^ ) HDL(r = −0.32, *p* = 0.000003) TCH (r = 0.23, *p* = 0.000985 ^b^)	HDL (r = −0.36, *p* = 0.000161)	-	Res (r = 0.33; *p* = 0.034394 ^b^) HDL (r = −0.41, *p* = 0.002373 ^b^) TCH (r = 0.42, *p* = 0.001712 ^b^)	HDL (r = −0.23, *p* = 0.005725 ^b^) TCH (r = 0.18, *p* = 0.030878 ^b^)
	CC	Glucose (r = 0.26, *p* = 0.001280 ^b^) HDL (r = −0.38, *p* = 0.000000) TCH (r = 0.19, *p* = 0.002289 ^b^)	Glucose (r = 0.32, *p* = 0.004972 ^b^) HDL (r = −0.32 *p* = 0.003608 ^b^)	-	HDL (r = −0.39, *p* = 0.002754 ^b^)	Glucose (r = 0.30, *p* = 0.003431 ^b^) HDL (r = −0.38, *p* = 0.000038) TCH (r = 0.23, *p* = 0.011609 ^b^)
HOMA-IR	Both	TG (r = 0.15, *p* = 0.010409 ^b^) Res (r = 0.40, *p* = 0.000000)	Res (r = 0.49, *p* = 0.000000) HDL (r = −0.19, *p* = 0.025190 ^b^)	Res (r = 0.31, *p* = 0.000470)	Res (r = 0.50, *p* = 0.000001)	Res (r = 0.41, *p* = 0.000000)
	GG + CG	Res (r = 0.32, *p* = 0.000144)	Res (r = 0.37, *p* = 0.000979)	Res (r = 0.27, *p* = 0.039224 ^b^)	Res (r = 0.48, *p* = 0.001436 ^b^)	Res (r = 0.35, *p* = 0.000824)
	CC	Res (r = 0.49, *p* = 0.000000)	Res (r = 0.62, *p* = 0.000000)	Res (r = 0.39, *p* = 0.001541 ^b^)	Res (r = 0.52, *p* = 0.000184)	Res (r = 0.49, *p* = 0.000013)

Res: resistin; Ins: insulin; HOMA-IR: homeostatic model assessment-insulin resistance; HDL: low-density lipoprotein; TG: triglycerides; TCH: total cholesterol. * Spearman rank correlation, r: correlation coefficient (rho). Correlations of HOMA-IR value with glucose and insulin are not shown. ^b^ Not significant after Bonferroni correction (*p* > 0.00092).

## Data Availability

Not applicable.

## Supplementary Materials

The following supporting information can be downloaded at: https://www.mdpi.com/article/10.3390/nu14235092/s1. Figure S1. Flow diagram of the study; Table S1. Multivariable logistic regression models estimated to evaluate the risk of hypertriglyceridemia in relation to the studied resistin variant.
