# Supplementary material for: The Relation between Resistin (−420C/G) Single Nucleotide Variant, Resistin Serum Concentration, Carbohydrate, and Lipid Parameters and Fried Food Taste Preference in Patients with Hypertriglyceridemia
Source: Nutrients. 2022 Dec 1;14(23):5092. doi: 10.3390/nu14235092 (PMC9738212; doi:10.3390/nu14235092)
Supplement: Supplementary file 1 [file nutrients-14-05092-s001.zip › nutrients-2002485-supplementary.pdf]

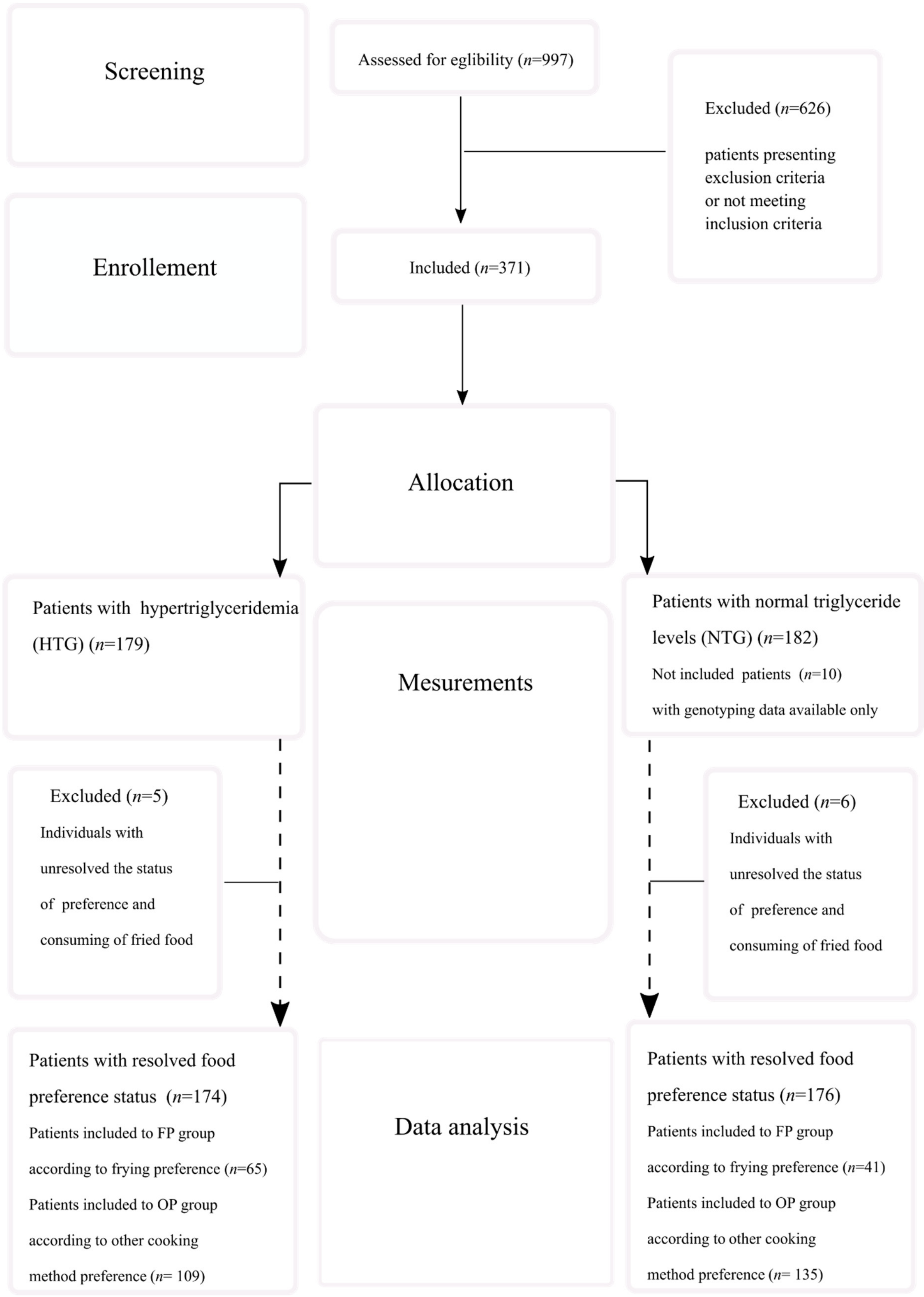

Figure S1. Flow diagram of the study.

HTG: hypertriglyceridemia; NTG: normotriglyceridemia; FP: frying preference; OP: other cooking preferences

Table S1. Multivariable logistic regression models estimated to evaluate the risk of hypertriglyceridemia in relation to the studied resistin variant

				**Model 1**			
**variables**		**raw 0R**	**95%CI**	**p**	**adjusted OR**	**95%CI**	**p**
sex							
female				*reference*			
male		1.693	1.096-2.615	0.018	2.088	1.246-3.498	0.005
genotype							
	CC			*reference*			
	CG+GG	1.296	0.860-1.952	0.216	1.747	1.085-2.812	0.022
BMI		0.977	0.932-1.025	0.342	0.962	0.911-1.016	0.167
age		1.017	0.999-1.036	0.065	1.016	0.995-1.036	0.129
				**Model 2**			
sex							
	female			*reference*			
	male	1.693	1.096-2.615	0.018	1.604	0.999-2.575	0.055
genotype							
	CC			*reference*			
	CG	1.391	0.899-2.152	0.139	1.718	1.056-2.795	0.029
	GG	1.152	0.599-2.216	0.672	1.078	0.538-2.162	0.832
fried food preference		2.006	1.262-3.187	0.003	1.954	1.188-3.212	0.008
total cholesterol [mg/dL]		1.003	1.003-1.007	0.061	1.004	1.000-1.007	0.063
				**Model 3**			
sex							
	female			*reference*			
	male	1.693	1.096-2.615	0.018	2.483	1.353-4.559	0.003
genotype							
	CC			*reference*			
	CG	1.391	0.899-2.152	0.139	1.930	1.053-3.538	0.033
	GG	1.152	0.599-2.216	0.672	1.118	0.467-2.675	0.802
fried food preference		2.006	1.262-3.187	0.003	1.464	0.811-2.643	0.205
HOMA-IR		1.165	0.055-0.997	0.055	1.246	1.010-1.537	0.040
total cholesterol [mg/dL]		1.003	1.003-1.007	0.061	1.003	0.999-1.007	0.135

HOMA-IR: homeostatic model assessment-insulin resistance; BMI: body mass index; p—statistical significance; OR—odds ratio; CI- confidence interval.
